# Multi‐Tissue Integrated Tissue‐Engineered Trachea Regeneration Based on 3D Printed Bioelastomer Scaffolds

**DOI:** 10.1002/advs.202405420

**Published:** 2024-08-19

**Authors:** Xingqi Song, Peiling Zhang, Bin Luo, Ke Li, Yu Liu, Sinan Wang, Qianyi Wang, Jinyi Huang, Xiaohong Qin, Yixin Zhang, Guangdong Zhou, Dong Lei

**Affiliations:** ^1^ Department of Plastic and Reconstructive Surgery Department of Cardiology Shanghai Key Lab of Tissue Engineering Shanghai 9th People's Hospital Shanghai Jiao Tong University School of Medicine Shanghai 200011 P. R. China; ^2^ College of Textiles State Key Laboratory for Modification of Chemical Fibers and Polymer Materials Donghua University Shanghai 201620 P. R. China

**Keywords:** 3D printing, bioelastomer, cartilage, tissue engineering, trachea regeneration

## Abstract

Functional segmental trachea reconstruction is a critical concern in thoracic surgery, and tissue‐engineered trachea (TET) holds promise as a potential solution. However, current TET falls short in fully restoring physiological function due to the lack of the intricate multi‐tissue structure found in natural trachea. In this research, a multi‐tissue integrated tissue‐engineered trachea (MI‐TET) is successfully developed by orderly assembling various cells (chondrocytes, fibroblasts and epithelial cells) on 3D‐printed PGS bioelastomer scaffolds. The MI‐TET closely resembles the complex structures of natural trachea and achieves the integrated regeneration of four essential tracheal components: C‐shaped cartilage ring, O‐shaped vascularized fiber ring, axial fiber bundle, and airway epithelium. Overall, the MI‐TET demonstrates highly similar multi‐tissue structures and physiological functions to natural trachea, showing promise for future clinical advancements in functional TETs.

## Graphical abstract

This study introduces a novel Multi‐tissue Integrated Tissue‐Engineered Trachea (MI‐TET) based on 3D printed bioelastomer scaffolds. Through 2D patterning and 3D assembling techniques, the researchers effectively organize several kinds of cells to create a well‐structured TET. This TET included C‐shaped cartilage rings, O‐shaped vascularized fiber rings, axial fiber bundle, and airway epithelium, closely mimicking the architecture of natural trachea, which holds promising implications for clinical applications in the repair of segmental trachea defects.

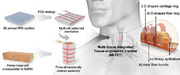

## 1 Introduction

Repairing segmental tracheal defects has long been a significant challenge in clinical practice due to the lack of ideal tracheal substitutes. Patients with extensive tracheal defects following tracheostomy often require substitute implantation for tracheal reconstruction. Current options include artificial tracheal prostheses, autologous or allogeneic tissue, and allogeneic tracheal grafts.^[^ ^1^ ^]^ However, these substitutes have shortcomings in structure, function, and biocompatibility, which can impact the outcome of tracheal reconstruction. Tissue engineering is a groundbreaking field in medicine that has shown promise in providing tracheal grafts with specific functions for segmental trachea reconstruction.^[^ ^2^ ^]^ The development of TETs with bio‐activity and physiological function is of great clinical significance.^[^ ^3^, ^4^ ^]^ However, the complex biological structures and functional characteristics of the trachea pose a significant challenge in TET reconstruction.^[^ ^5^ ^]^ Physiologically, the trachea consists of C‐shaped cartilage rings (C ring), O‐shaped vascularized fiber rings (F ring), dorsal axial fiber bundle (F bundle), and inner airway epithelium.^[^ ^6^ ^]^ This unique multi‐tissue structure allows the trachea to adapt to the mechanical stresses of respiration and movement. Successful TET reconstruction hinges on regenerating heterogeneous tissues and achieving biomimetic regeneration of cartilage, blood vessels, and airway epithelium.^[^ ^7^ ^]^ Despite numerous studies on TETs, an optimal construction strategy to simultaneously achieve chondrogenesis, vascularization, and epithelization is still lacking, leading to unsatisfactory clinical outcomes.

In recent decades, several studies have developed single‐cartilage tubular TET using various bio‐materials. However, the clinical application of these biological or synthetic materials requires further extensive experimentation to confirm their safety.^[^ ^8^, ^9^, ^10^, ^11^, ^12^ ^]^ Acellular matrix is considered a suitable scaffold material for TET due to its preservation of natural extracellular matrix components and micro‐structure. Elliott et al. utilized an acellular allogeneic trachea as a scaffold, seeded with autologous bone marrow mesenchymal stem cells and nasal mucosal epithelial cells, to address a segmental tracheal defect in a pediatric case. Unfortunately, the patient passed away 15 days post‐surgery, and bronchoscopy revealed significant narrowing of the tracheal lumen.^[^ ^13^ ^]^ Additionally, Sun et al. developed an acellular cartilage matrix hydrogel for tracheal cartilage regeneration, but encountered challenges related to limited sources of acellularized matrix and the inability to replicate various tissue structures of the trachea.^[^ ^14^ ^]^ In a previous study, an assembly technique involving alternating cartilage rings and polymer rings (cell‐free) was introduced to closely imitate the cartilage/fiber‐alternating structure of the trachea.^[^ ^10^ ^]^ Another investigation successfully regenerated a neo‐trachea using C‐shaped cartilage, which closely resembled the mechanical characteristics of the natural trachea.^[^ ^15^ ^]^ However, issues related to epithelialization were overlooked.

Nowadays, the significant impact of 3D printing technology on innovation and advancements in regenerative medicine is evident, particularly in its capacity to tailor macro‐forms and control micro‐structures with precision.^[^ ^16^ ^]^ Various studies have explored the application of 3D printing technology in achieving TET regeneration.^[^ ^17^, ^18^, ^19^, ^20^ ^]^ Our laboratory recently introduced a novel approach for segmental trachea reconstruction using 3D‐bioprinted biomimetic trachea composed of two distinct tissue‐specific matrix hydrogels for cartilage and fibrous tissue regeneration.^[^ ^7^, ^21^ ^]^ Despite these advancements, several challenges persist, including the need for a precisely designed and naturally bionic alternant structure between C rings and F rings, reliance on random cell infiltration for connective tissue regeneration leading to unstable tissue formation and inadequate vascularization, and the absence of mucosal epithelium in the regenerated trachea. Addressing these complexities, particularly in achieving a multi‐tissue structured tracheal equivalent, remains a significant challenge.

Bioelastomers are biocompatible and biodegradable materials, making them crucial in the field of biological materials and a prominent area of research in recent years. Their desirable ability to recover from deformation makes them suitable for simulating the mechanical properties of the trachea.^[^ ^22^, ^23^, ^24^ ^]^ Poly glycerol sebacate (PGS), a representative of bioelastomer, has gained remarkable results in wide areas such as myocardial, blood vessel, cartilage, and retinal tissue engineering.^[^ ^25^, ^26^ ^]^ Despite its FDA approval for clinical use in 2021,^[^ ^27^ ^]^ the long cross‐linking process required for PGS poses a challenge for integration with 3D printing methods. Our previous research introduced a novel salt‐composite‐printing strategy to successfully 3D print PGS, leading to the development of chondrogenic bioactive PGS scaffolds for efficient cartilage regeneration.^[^ ^24^, ^28^, ^29^ ^]^ We further innovated by using 4‐axis 3D printing to create tubular PGS scaffolds with adjustable braid structure, resulting in a bionic tubular tracheal cartilage with excellent mechanical elasticity.^[^ ^30^ ^]^ However, the mechanical properties of individual tubular cartilage structures may be excessively rigid. When using a 3D printed PGS scaffold for] cartilage regeneration, the precise construction of the alternating C rings and F rings poses a challenge. Furthermore, integrating with airway epithelium for optimal functionality becomes even more complex.

**Figure 1:**
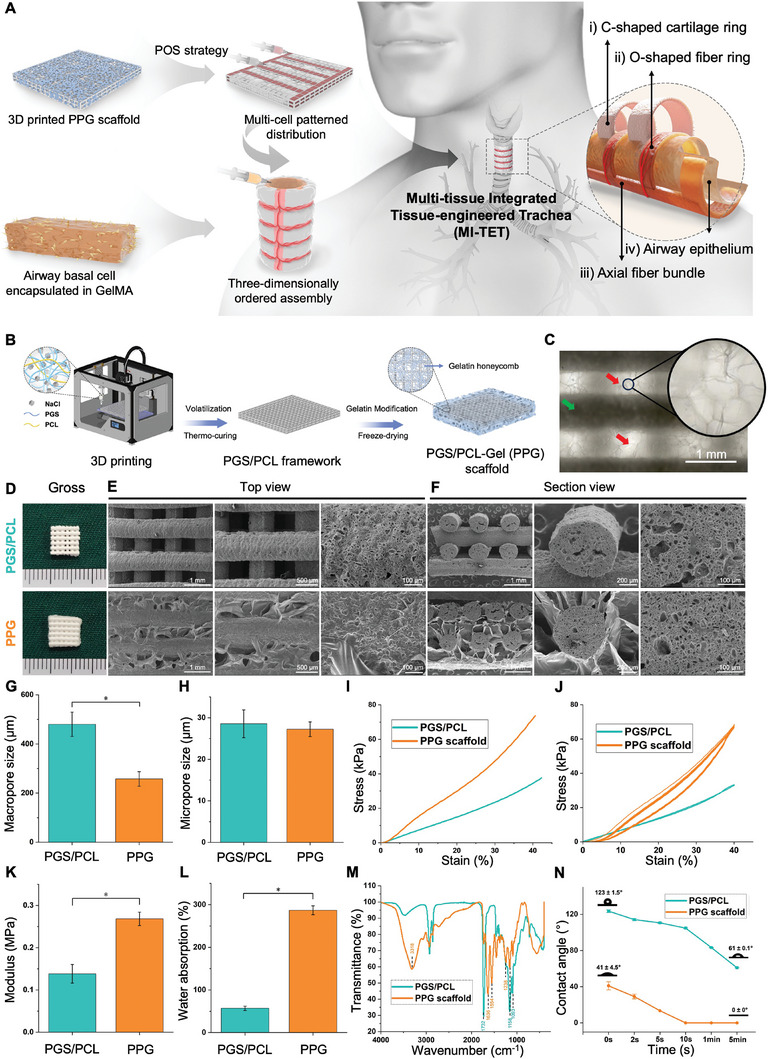
The overall research design and characterization of the PGS/PCL‐Gelatin (PPG) scaffolds. A) Schematic illustration for the design of MI‐TET. B) Fabrication process of the PPG scaffolds. 3D‐printed and thermo‐cured PGS/PCL scaffolds were composited with gelatin fibrous network to fabricate PPG scaffolds. C) Optical microscope images of the PPG scaffolds. Green and red arrows represent PGS/PCL framework and gelatin fibrous network, respectively. Scale bars: 1 mm. D) Gross images of 3D‐printed PGS/PCL scaffolds and PPG scaffolds. E,F) SEM images of the two scaffolds at different magnifications in top view and section view. Scale bars: 1 mm, 500 µm, 200 µm, 100 µm. G) Macropore size of the PGS/PCL framework and gelatin fibrous network, *p < 0.05. H) Micropore size inside the filaments. I) Typical compressive stress–strain curves resulting from uniaxial compression tests. J) Cyclic compression tests for ten cycles with maximum strain of 40%. K) Comparison of compressive modulus, *p < 0.05. L) Water absorption rate, *p < 0.05. M) FTIR spectroscopy analysis. N) Dynamic water contact angle changing curve with time.

To overcome the aforementioned obstacles, we successfully designed and constructed a multi‐tissue integrated tissue‐engineered trachea (MI‐TET) using 3D‐printed PGS scaffolds to mimic the complex structure and physiological functions of the natural trachea. **Figure** **1**A depicts the concept and design progress: we utilized 3D printed PGS/PCL‐Gelatin (PPG) bioactive scaffolds and a post‐occupancy sacrifice (POS) strategy with a thermosensitive hydrogel (Pluronic F127) to achieve a two‐dimensional patterned heterogeneous construction of chondrocytes and fibroblasts. A subsequent 3D assembly strategy achieved the orderly 3D regeneration of C rings/F rings alternating structures and F bundle in vivo, closely resembling the physiological architecture of the natural trachea. For the bionic airway epithelium, we employed photo‐crosslinked gelatin methacryloyl hydrogel (GelMA) to load cultured airway basal cells isolated from tracheal mucosa onto the inner wall of the regenerated trachea, resulting in functional biomimetic airway epithelium. Overall, this MI‐TET achieves the regeneration of a four‐fold tissue structure with similar elastic strength and tissue structure to the natural trachea, indicating its potential as a promising method for clinically constructing functional trachea in future tissue engineering.

## 2 Results and Discussion

### 2.1 Preparation and Characterization of the PPG Scaffolds

In our previous study, we successfully employed a salt‐composite‐printing strategy to achieve the 3D printing of the bioelastomer PGS for the first time.^[^ ^30^, ^31^ ^]^ In this study, we constructed the PGS/PCL‐Gelatin (PPG) hierarchical microporous scaffolds as showed in Figure 1B. Both PGS and PCL polymer contain ester bonds, susceptible to degradation through hydrolysis. PGS is a rapidly degradable bioelastmor, and excessive degradation leads to poor structural stability during tissue regeneration.^[^ ^22^ ^]^ PCL is a stiff biomaterial with a slow degradation rate (in vivo degradation time exceeds 1 year), which leads to long‐term residual scaffold during tissue regeneration.^[^ ^32^ ^]^ Our previous study has shown that the addition of PCL can significantly reduce the degradation rate of PGS and improve the structural stability of the scaffold during the degradation process.^[^ ^33^ ^]^ Due to the rapid degradability and softness of PGS,^[^ ^29^ ^]^ we printed the scaffolds by mixing PGS and PCL to slow down the degradation and improve mechanical support of PGS scaffolds, to ensure structural stability of trachea regeneration. PGS/PCL pre‐polymer and NaCl composite ink were 3D printed into multi‐layer constructs, which were then crosslinked in vacuum to obtain stable thermosetting PGS/PCL composite scaffolds (Movie S1, Supporting Information). To enhance the cell activity and inoculation efficiency of PGS/PCL scaffolds, the 3D printed scaffolds were immersed in 1% gelatin solution and transformed into 3D porous freeze‐dried scaffolds using a vacuum freeze dryer for 24 h. This process resulted in the formation of a gelatin network with small pore size (Gelatin honeycomb) within the PPG scaffolds (Figure 1C). To improve stability and mechanical properties, the PPG scaffolds were cross‐linked using EDC/NHS reaction for 24 h. The residual solution was completely removed in deionized water, and finally, the PPG scaffolds were obtained through vacuum freeze‐drying. Eventually, PPG scaffolds finished Gelatin modification and still maintained the initial morphological features of the PGS/PCL scaffolds, with gelatin evenly filled into the interstitial areas (Figure 1D). SEM images demonstrated that gelatin was present throughout and integrated into the PGS framework with well‐organized hierarchical structures (Figure 1E,F). A multi‐layered PPG framework was created using vertically stacked filaments with a diameter of 700 µm approximately. In comparison to the macropore size of the PGS/PCL framework (480.12 ± 49.35 µm), gelatin fibrous networks were evenly distributed between PGS/PCL filaments in the PPG scaffolds, resulting in smaller macropores (257.60 ± 29.87 µm) (Figure 1G). During the cell seeding and in vitro culture, the cells adhered to and grew in the macropores of the scaffolds. The macropore size of the PPG scaffolds was more suitable for cell growth and proliferation, while the macropore size of PGS/PCL scaffolds was much larger (almost two times). Furthermore, there were plenty of cubic micropores obtained by salt leaching, existing on the surface and inside of 3D printed filaments. The micropore sizes of the PGS/PCL and PPG scaffolds were 28.58 ± 3.34 µm and 27.24 ± 1.78 µm, respectively, and they are determined by the salt particulate size, which could be adjusted by the grinding and sieving process during preparation (Figure 1H). These micropores provided channels for nutrient substance and extracellular matrix to migrate and accumulate in PGS/PCL filaments in vivo.^[^ ^28^, ^29^ ^]^

The addition of gelatin significantly improved the stiffness of the PGS/PCL scaffolds because of the hydrogen bond interactions of amino and hydroxyl groups on the molecular chain of the gelatin.^[^ ^34^ ^]^ The uniaxial compression tests established that the PGS/PCL and PPG scaffolds had an elastic modulus of 138.25 ± 21.97 kPa and 268.37 ± 15.85 kPa, respectively (Figure 1I,K). Furthermore, cyclic compressive tests determined that the PGS/PCL and PPG scaffolds had excellent elasticity and anti‐fatigue properties with limited hysteresis under dynamic pressure (Figure 1J). The elasticity and fatigue durability of the scaffolds enabled the regenerated cartilage tissue to withstand multiple dynamic deformations and maintain its original morphology after implantation. Furthermore, the modification of gelatin resulted in increased hydrophilicity of the PPG scaffolds. Upon water absorption, the weight increasing percentage of PPG scaffolds (286.95 ± 10.47%) was significantly higher than that of PGS/PCL scaffolds (56.95 ± 4.98%) (Figure 1L; Figure S1A, Supporting Information). The FTIR analysis (Figure 1M) further supports the enhanced hydrophilicity of PPG materials, as indicated by the characteristic absorption peak at 3318, 1636, 1554, and 1238 cm^−1^ corresponding to hydrophilic N─O, C─O, and amine groups, whereas the characteristic absorption peak at 1158 and 1732 cm^−1^ of PGS/PCL scaffolds represents hydrophobic ester bonds. Additionally, in the water contact angle experiment (Figure 1N), the contact angle between PPG scaffolds and water droplet reached 0 within 10 s (complete absorption) (Movie S2, Supporting Information), while the contact angle in the PGS/PCL scaffolds group remained at 61° even after 5 min (incomplete absorption) (Movie S3, Supporting Information). These findings suggest that gelatin‐modified PPG scaffolds exhibit superior hydrophilicity and biological activity, making them more suitable for various cell behaviors, including penetration, adhesion, proliferation, and matrix secretion.

### 2.2 2D Patterned Construction of the MI‐TET In Vitro

**Figure 2:**
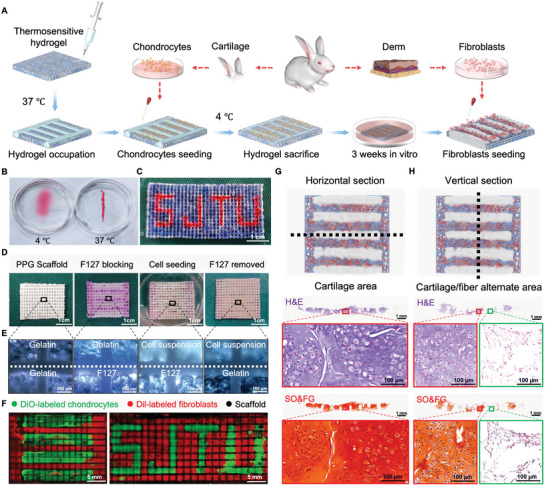
2D‐patterned construction of the MI‐TET in vitro. A) Schematic representation of the 2D‐patterned construction process in vitro using Post‐Occupancy Sacrifice (POS) strategy. B) The state of thermosensitive hydrogel (Pluronic F127) at 4 and 37 °C. C) The precise patterned distribution of Pluronic F127 on PPG scaffolds. Scale bars: 1 cm. D) The process of multi‐cellular patterned construction on PPG bioactive scaffolds using the POS strategy. Scale bars: 1 cm. E) Optical images during the process of cell patterned seeding. Scale bars: 500 µm. F) Distribution map of tracheal derived and “SJTU” abbreviation patterns, which were taken under confocal microscope after fluorescent live cell staining of cells. Green region represents DiO‐labeled chondrocytes, red region represents Dil‐labeled fibroblasts, and black region represents filaments of PPG scaffolds. Scale bars: 5 mm. G,H) Histological staining results of patterned multicellular scaffold complex after 4 weeks in vitro culture (HE and SO/FG staining on the horizontal and vertical section of regenerated tissue at 4 weeks in vitro). Scale bars: 1 mm, 100 µm.

In order to address the issue of the intricate spatial interweaving between the C rings and F rings of the trachea, as well as the F bundle structure, we first developed a Post‐Occupancy Sacrifice (POS) strategy of thermosensitive hydrogel to facilitate a patterned construction of multiple cells. We selected Pluronic F‐127 with concentration of 30% (wt.%), which exhibits a thermosensitive property–being liquid at 4 °C and gelatinous semi‐solid at 37 °C^[^ ^35^ ^]^ (**Figure** **2**B). This gelatinous semi‐solid can effectively impede the distribution of cells on our PPG scaffolds, and subsequently transition into liquid state and dissipate at 4 °C, so that it plays a role of temporary obstructive barrier during cell seeding process. Through this POS strategy, the precise distribution of various complex patterns can be easily controlled (Figure 2C), and it helps achieve accurate patterning distribution construction on our PPG scaffolds (Figure 2D).

Figure 2A illustrates the step‐by‐step process of multi‐cellular patterning distribution on the PPG scaffolds. Initially, a thermo‐sensitive hydrogel, Pluronic F‐127, formed a semi‐solid hydrogel at a high temperature (37 °C) and effectively occupied a specific position on the PPG scaffolds. Subsequently, chondrocyte suspension was seeded in the unoccupied areas, resulting in precise distribution of multi‐strips structure. Following this, the Pluronic F‐127 was liquefied and eliminated at low temperature (4 °C), leaving behind the chondrocytes orderly arranged in strips. This arrangement served as the foundation for the subsequent construction of C ring in a 3D assembly. After culturing the cartilage tissue in vitro for 3 weeks, fibroblasts were introduced into the remaining positions, enabling the spatial cross‐distribution of the C rings and F rings.

In order to verify the accuracy of our approach, we conducted several validation tests from three perspectives: optical microscopy, fluorescent cell labeling, and histological staining. During the cells seeding process, we captured microscopic images at each step using an optical microscope with an external light source (Figure 2E). These images clearly showed the distinct boundaries among gelatin, Pluronic F‐127, and the cell suspension on the PPG scaffolds. This indicated that our POS strategy effectively enabled the patterned distribution of the cell suspension. To further confirm the accuracy of the patterned construction, we stained chondrocytes and fibroblasts with two different live cell fluorescent dyes (DiO (green) and Dil (red)) and then seeded them onto the PPG scaffolds using POS strategy as before (Figure 2F). Green region represents DiO‐labeled chondrocytes, red region represents Dil‐labeled fibroblasts, and black region represents filaments of scaffolds. Under the fluorescence microscope, the two fluorescently labeled cells showed clear patterned distribution, which demonstrates that our POS strategy have achieved precise multicellular distribution on PPG scaffolds. After culturing the scaffold‐cell complex in vitro for 4 weeks (3 weeks for chondrocytes only and 1week for chondrocytes and fibroblasts), we then verified the accuracy through HE (Hematoxylin and Eosin) (Figure 2G) and SO/FG (Safranine‐O and Fast Green) (Figure 2H) staining in transverse and longitudinal sections respectively. In the transverse section, the cartilage tissue in the area of continuous chondrocytes seeding appeared to be mature, with a continuous and uniform distribution. The chondroid tissue also showed initial regeneration, characterized by the presence of typical lacunae structures unique to cartilage and accompanied by extracellular matrix deposition. In the longitudinal section, the area where chondrocytes and fibroblasts were alternately seeded still maintained the alternating distributional structure. Overall, these results indicate that the POS strategy of thermosensitive hydrogel was effective in achieving multi‐cellular patterned construction, and demonstrated the biomimetic spatial structure in the MI‐TET during the in vitro culture stage.

### 2.3 3D Regeneration of the MI‐TET for 8 and 12 weeks In Vivo

**Figure 3:**
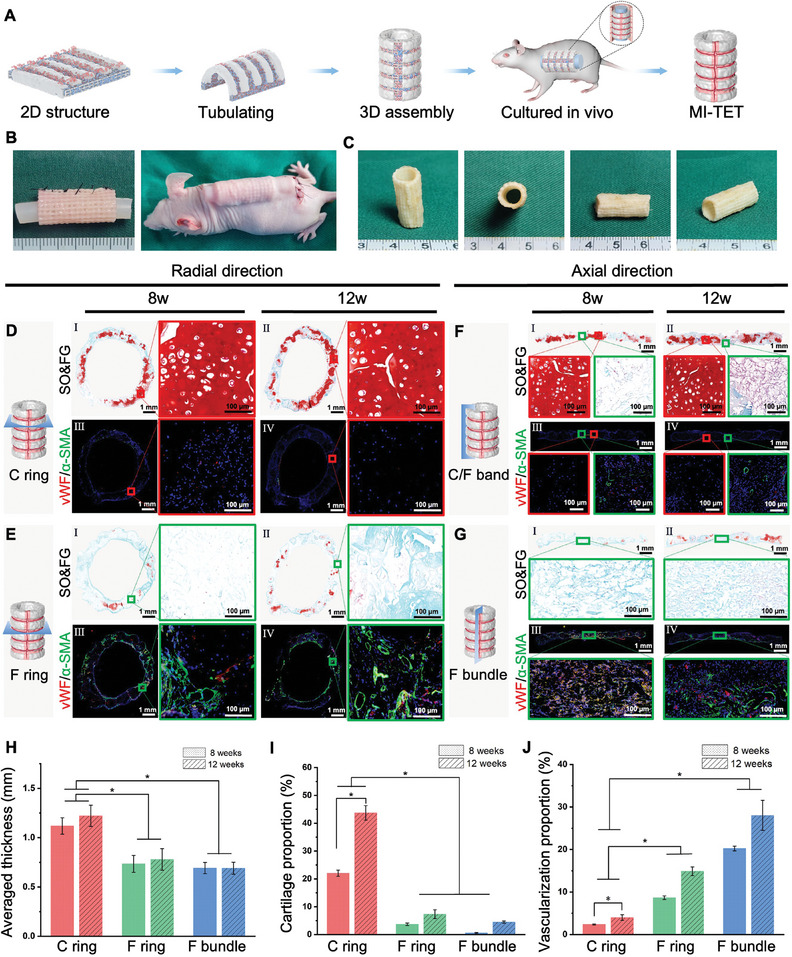
Regeneration of the MI‐TET in nude mice for 8 and 12 weeks. A) Schematic illustration of the MI‐TET regeneration via 3D assembly strategy. B) General morphology of the MI‐TET before and during subcutaneous implantation in nude mice. C) General morphology of the MI‐TET after subcutaneous culture in nude mice. D–G) Results of SO/FG and vWF and *α*‐SMA staining of 8/12w‐in vivo regenerated trachea in four sections: C ring section and F ring section in radial direction; C/F band and F bundle section in axial direction. I/II represents the SO/FG staining results corresponding to 8 and 12 weeks. III/IV represents the vWF (Red) and *α*‐SMA (Green) staining results corresponding to 8 and 12 weeks. Red rectangles represent cartilage regions; green rectangles represent vascularized fibrous tissue regions; Scale bar: 1 mm, 100 µm. Statistical map of H) averaged thickness, J) cartilage proportion and K) vascularization proportion of C ring, F ring and F bundle at 8/12 weeks, *p < 0.05.

After realizing the patterned structure on 2D plane, we further developed 3D MI‐TET. We rolled up the scaffold‐cell complex in vitro and implanted them under the skin of nude mice, with the silica‐gel rod in the middle providing internal support to prevent collapse of the regenerated trachea due to skin tension (**Figure** **3**A,B) (Movie S4, Supporting Information). After 8 and 12 weeks of in vivo culture, the regenerated trachea tissues were extracted. The regenerated trachea maintained the original tubular structure, with the internal pores of the scaffold gradually filled with neo‐tissues. The tracheal structures consisted of porcelain white C rings and F rings, which were alternately distributed (Figure 3C).

To evaluate the maturity of the implanted tracheal cartilage and determine if the multi‐tissue patterned construction could be maintained after in vivo culture, histological staining was performed on two sections of the radial and axial sections of the MI‐TET. The radial section corresponded to the C ring and F ring of the trachea respectively, while the axial section corresponded to the longitudinal cartilage/fiber interleaving area (C/F band) and the F bundle respectively. Figure 3D–G showed the SO and FG staining (I/II) and vaso‐specific immunofluorescence staining of vWF (Von Willebrand factor) and *α*‐SMA (*α* smooth muscle actin) (III/IV) in these four sections at 8 weeks and 12 weeks. The vWF (Red) represents neo‐vessels and the *α*‐SMA (green) represents mature vessels. In SO and FG staining, the cartilage tissue area exhibited the presence of mature cartilage with a typical lacunar structure and a significant amount of extracellular matrix (ECM) deposition. The cartilage maturity at 12 weeks was significantly higher than that at 8 weeks. The cartilage matrix basically presented a C‐shaped distribution, which was consistent with the patterned construction in vitro (Figure 3D). In contrast, the fibrous tissue area was predominantly covered by fibrous tissues almost without distribution of cartilage tissue, especially in F ring and F bundle sections (Figure 3E,G). Although there was a small amount of chondrocyte expression in the fibrous ring and fibrous band regions, the axial C/F band still maintained the alternating arrangement of cartilage tissue and fiber tissue (Figure 3F), due to the migration and fusion of adjacent chondrocytes and fibroblasts in the PPG porous scaffold. The integration of tissue interfaces did not disrupt the alternating structure of cartilage and fibers, but rather helped to improve the mechanical strength of MI‐TET.

Angiogenesis could only be observed exclusively in the fibrous tissue area, rather than cartilage area, so we can also verify the cartilage/fiber distribution by specific staining of vascularization indicators. The vaso‐specific vWF and *α*‐SMA immunofluorescence staining revealed that the positive region was primarily confined to the fibrous tissue region, that is, the F ring, F bundle, and fiber area in the C band and C/F band (Figure 3D–G. III/IV). Additional staining techniques including HE (Figure S3, Supporting Information), Masson (Figure S4, Supporting Information), and type II collagen (Figure S5, Supporting Information) staining further confirmed the formation of chondroid tissue in the radial C ring area, which exhibited typical lacunar‐like structures and extensive ECM deposition. The axial C/F band still maintained the alternating arrangement of cartilage tissue and fiber tissue. The section of the F bundle was mostly covered by fiber tissue.

Quantitatively we selected C ring (red), F ring (green), and F bundle (blue), and analyzed the thickness, cartilage proportion and vascularization proportion of these three sections at 8 and 12 weeks separately. The tracheal thickness in the cartilage area was significantly greater than in the other two fiber areas (Figure 3H). Additionally, Figure 3I demonstrated that the cartilage ring area had a much higher proportion of cartilage compared to the other two fiber regions, and it increased with the time of culture. This observation is consistent with the maturity of cartilage tissue and the deposition of ECM. Analysis of the vascularization immunofluorescence results for these three areas (Figure 3J) indicated that the degree of vascularization was significantly greater in the F ring and axial F bundle compared to the C ring. Therefore, our MI‐TET successfully maintained the original structural design in nude mice.

In previous studies, it was found that single cartilage trachea using 4‐axis 3D printed PGS scaffolds had relatively poor cartilage thickness and structural integrity.^[^ ^30^ ^]^ This may be attributed to a design defect, as it lacked vascularized fibrous rings and cartilage inherently lacks blood vessels, resulting in inadequate nutrient supply to the trachea. On the other hand, our MI‐TET with an alternating structural design of cartilage/fiber ensured that the cartilage ring received sufficient nutrients from the adjacent vascularized fiber rings. This was a crucial factor contributing to the more superior regenerated cartilage observed in our MI‐TET. Currently, we have successfully achieved the bionic integration of three levels: C ring, F ring, and F bundle.

### 2.4 Biomimetic Construction of the Airway Epithelium

The bionic construction of airway epithelium aims to replicate the natural structure and function of the respiratory tract. Airway basal cells, located in the basal layer of airway epithelium, are pluripotent stem cells that have the capacity to self‐replicate and differentiate into different cell types, including epithelial cells, mucus cells, and ciliary cells, etc. These cells play a vital role in the growth and repair of respiratory epithelial cells.^[^ ^36^, ^37^, ^38^ ^]^ Additionally, airway basal cells are involved in immune and inflammatory responses of respiratory cells, as well as processes like bronchial hyperplasia and airway remodeling.^[^ ^39^ ^]^ The relationship between airway basal cells and respiratory diseases such as asthma, chronic obstructive pulmonary disease, and bronchiectasis has gained significant attention in recent years, making it an important area of research in the field of respiratory diseases.^[^ ^40^, ^41^ ^]^

**Figure 4:**
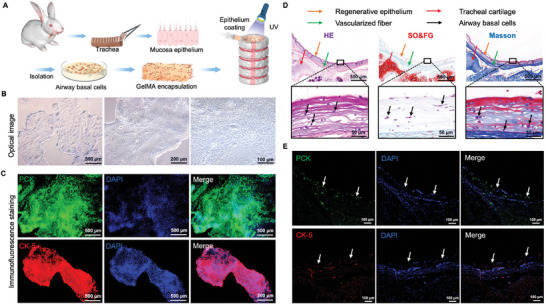
Airway basal cells culture and epithelium construction of MI‐TET. A) Schematic illustration for the construction process of airway epithelium regeneration. B) Optical microscope images and C) immunofluorescence images of PCK and CK‐5 for airway basal cells. Scale bar: 500, 200, 100 µm. D) Histological examinations of regenerated airway epithelium (Orange arrows). Red arrows show cartilage, green arrows show fiber texture, black arrows show airway basal cells. Scale bar: 500, 50 µm. E) Immunofluorescence staining of PCK and CK‐5 for regenerated airway epithelium. White arrows show airway basal cells. Scale bar: 100 µm.

In this study, we successfully isolated and extracted primary airway basal cells from the mucosa of rabbit trachea and rapidly expanded them in vitro. We utilized photo‐crosslinked GelMA hydrogel to load these airway basal cells onto our regenerative MI‐TET's inner wall (**Figure** **4**A), which allowed us to preliminarily achieve the bionic construction of the airway epithelium. Figure 4B illustrated the morphology and structure of the isolated and amplified airway basal cells observed at different magnifications under optical microscope. During in vitro culture, the airway basal cells form a single layer on the surface of the medium and continue to divide, proliferate, and produce new cells, and secrete mucus to protect the epithelial cells. To validate the reliability of the cell extraction and culture process, immunofluorescence staining of PCK (pan‐cytokeratin) and epithelial‐specific CK‐5 (cytokeratin‐5) were performed (Figure 4C). The staining results confirmed the high purity and cell viability of the cultured airway basal cells.

In order to validate the effectiveness of the airway epithelial construction, we conducted histological staining (HE, SO/FG and Masson staining) (Figure 4D) as well as immunofluorescence staining (PCK and CK‐5) in radial section (Figure 4E). The results demonstrated that a ring structure containing airway basal cells was observed on the inner wall of the regenerative trachea, confirming the effective regeneration of airway mucosal epithelium. At this point, we have achieved the integration of four structures in our biomimetic MI‐TET: C ring, F ring, F bundle, and airway epithelium inside the trachea.

### 2.5 Comprehensive Evaluation of the MI‐TET

**Figure 5:**
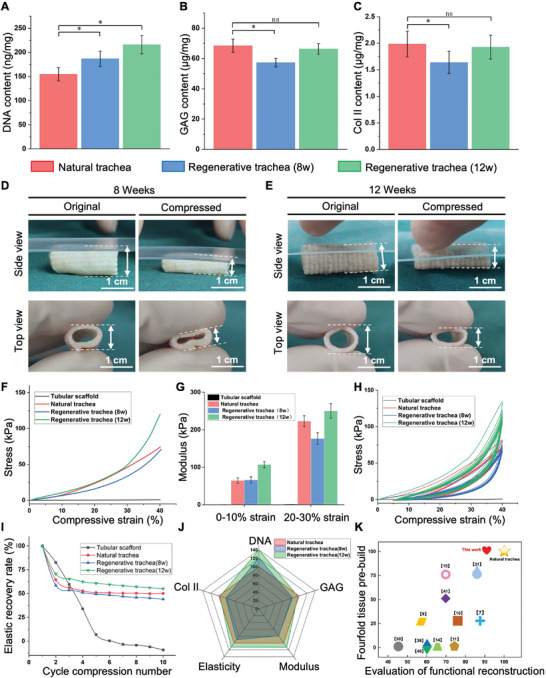
Comprehensive evaluation of the MI‐TET. A‐C) Quantitative analyses of the DNA contents, GAG contents, COL II among 8/12 week‐regenerated trachea and natural trachea, n = 4, *p < 0.05, ns: no significance. D,E) Morphological comparison of MI‐TET in original and compressed state from side and top view. Scale bar: 1 cm. F) Stress–strain curves resulting from uniaxial compression tests. G) Column diagram of modulus corresponding to compressive strain during 0–10% and 20–30%. H) Cyclic compression tests with maximum strain of 40%. I) The elastic recovery rate curve with the number of cyclic compressions in the first ten cycles. Elastic recovery rate is the ratio of rebound stress to compressive stress in the same compression test. J) A comprehensive evaluation chart from five dimensions of DNA, GAG, Col II, elastic modulus and elastic recovery rate. K) Scatter plot of TET regeneration studies in past decade based on functional and structural reconstruction effect of regenerated trachea. The data used are summarized in Table S1 (Supporting Information).

In this chapter, we present a comprehensive evaluation of our MI‐TET, comparing it with the natural trachea through quantitative analysis and elastic testing. For the quantitative analysis of tissue composition, we determined the DNA content (**Figure** **5**A), GAG content (Figure 5B), and type II collagen content (Figure 5C) of the MI‐TET cultured for 8 and 12 weeks, and compared them with natural trachea. The results revealed that the DNA content of the regenerated tracheal cartilage was higher due to the high‐density cell suspension seeding. Over time, as chondrocytes secreted ECM, the content of GAG and type II collagen in the regenerated trachea increased and gradually approached the level in natural tracheal cartilage.

By squeezing the MI‐TET, we discovered that they demonstrated similar elasticity and rigidity to natural trachea. Notably, the mechanical properties of the regenerated trachea at 12 weeks showed significant improvement compared to those at 8 weeks (Figure 5D,E) (Movies S5 and S6, Supporting Information). To analyze these mechanical properties in more detail, we conducted uniaxial compression and cyclic compression tests. The compressive stress–strain curve observed during a single compression process is illustrated in Figure 5F. It was evident that the PPG tubular scaffolds alone lacked the necessary elastic and mechanical structure. Therefore, we performed calculations and comparisons of the elastic moduli of the other three tracheas at small (0–10%) and large (20–30%) strains (Figure 5G). Results revealed that under small strain conditions, the modulus of the regenerated trachea at both 8 and 12 weeks could match that of the natural trachea. However, under large strain conditions, only the modulus of the regenerated trachea at 12 weeks could reach the level of the natural trachea, while the modulus of the regenerated trachea at 8 weeks was obviously inadequate. Figure 5H illustrated the compressive stress‐strain curve during cyclic compressions. We analyzed the first ten cycles and determined the ratio of rebound stress to compressive stress at a compression strain of 20% in each cycle. This ratio, known as the elastic recovery rate,^[^ ^30^ ^]^ served as a measure of the organization's elasticity and ability to recover deformation (Figure 5I). The PPG scaffolds initially showed inadequate elastic recovery rate, but after an 8‐week regeneration period, the trachea closely resembled the natural trachea in terms of elastic recovery. Moreover, the elastic recovery rate of the regenerated trachea after 12 weeks exceeded that of the natural trachea, indicating superior elastic characteristics under the same deformation. Combining the results of the two mechanical tests above, our MI‐TET displayed enhanced mechanical and elastic properties compared to the natural trachea, so that they could effectively maintain compliance and demonstrate improved functionality in the dynamic mechanical environment in vivo.

Comprehensively, we selected DNA, GAG, Col II, elastic modulus, and elastic recovery rate as five scoring criteria to evaluate the performance of the MI‐TET comprehensively. For quantitative evaluation, the index of natural trachea was given a full score of 100 and we obtained a five‐dimensional evaluation scale as shown in Figure 5J. The scoring results revealed that the indexes of the regenerated trachea cultured in vivo for 8 weeks were comparable to those of the natural trachea. Furthermore, the mechanical properties of the regenerated trachea cultured in vivo for 12 weeks were even superior to those of the natural trachea. This demonstrated that the MI‐TET exhibited comprehensive properties that closely resemble those of the natural trachea.

Furthermore, in order to compare with the latest scientific research in tissue engineering for tracheal regeneration, we have reviewed 12 significant studies (including this work) from the past decade, using the natural trachea as a reference point.^[^ ^7^, ^9^, ^10^, ^11^, ^14^, ^15^, ^21^, ^30^, ^42^, ^43^, ^44^ ^]^ Through detailed examination and comparison of these studies, we evaluated each one based on functional and structural tissue reconstruction dimensions (refer to Tables S1 and S2, Supporting Information). Subsequently, a scatter graph was generated to visualize the findings (Figure 5K). In evaluating tracheal function, we focused on five key indicators: GAG, collagen, modulus, vascularization level, and epithelization degree. Our analysis of the research literature revealed varying scores across these indicators. While some studies excelled in basic properties such as GAG, collagen, and elastic modulus, others showed significant deficiencies in vascularization level and epithelialization degree, resulting in lower scores (Table S1, Supporting Information). This study on MI‐TET regeneration outperformed all previous tracheal regeneration studies in terms of tracheal function, approaching the functionality of a natural trachea. In the realm of four‐fold tissue structure construction, previous studies have given minimal attention to the intricate multi‐tissue composition of the trachea. Only a handful of papers have successfully engineered trachea tissue regeneration in vitro that aligns with the natural physiological structure of the trachea (Table S2, Supporting Information). This study, for the first time, achieved the integrated construction of a fourfold tissue structure in vitro, effectively mimicking the C ring, F ring, F bundle, and airway epithelium. This breakthrough introduces a novel concept for the future advancement of TETs.

## 3 Conclusion

The aim of this study was to create a regenerative trachea using tissue‐engineering methods, with the goal of offering an effective treatment option for clinical use. To address the challenge of replicating the trachea's precise stereochemical structure, we utilized a POS strategy and a 3D assembly approach. For the bionics of airway epithelium, airway basal cells expanded in vitro were placed on the inner wall using photo‐crosslinked GelMA hydrogel. Eventually, our MI‐TET successfully replicated the structural and functional characteristics of four key tissues: C‐shaped cartilage ring (C ring), O‐shaped vascularized fiber ring (F ring), axial strip fiber bundle (F bundle), and inner airway epithelium. These components closely resembled the natural trachea in both biological structure and physiological function. However, our study still had some limitations, including the lack of precision in the patterned distribution due to manual manipulation and the non‐conforming physiological structure of the epithelial cells embedded in GelMA. Despite these limitations, this research introduces a novel approach for accurately replicating a multi‐tissue integrated trachea, marking a significant advancement in for trachea regeneration, and indicating a promising clinical application for the repair of segmental trachea defects.

## 4 Experimental Section

### Materials

Glycerol (analytical grade, ≥ 99%), sebacic acid (analytical grade, ≥ 99%) were purchased from J&K Scientific Ltd. PCL (Mn 80 000 g mol^−1^, Sigma–Aldrich.), Type A gelatin, N‐(3‐Dimethylaminopropyl)‐N′‐ethyl‐carbodiimide hydrochloride (EDC) and N‐Hydroxy‐succinimide (NHS) were purchased from Aladdin (Shanghai, China). Salt particulates (sodium chloride, NaCl, 99.5%) were purchased from Macklin (Shanghai Macklin Biochemical Co., Ltd.). GelMA was purchased from EFL (Shanghai, China).

### Preparation of PGS/PCL/salt Composite Ink

In brief, polycondensation of an equimolar amount of glycerol and sebacic acid synthesized PGS prepolymer according to the previous report.^[^ ^45^ ^]^ Salt particles were first ground into smaller size and then sifted to get fine salts (≤ 38 µm). PCL and PGS prepolymer were dissolved in THF (50%, w/v) at weight ratios of 1:9. PCL/PGS solutions were mixed with salt particles at the 1:2 weight ratio (PCL/PGS: salt). The mixture was placed into oven at 70 °C for 4 h to remove most of solvents. The resulted paste‐like mixture could be used as composited ink for 3D printing. To solve the problems of rapid degradation and insufficient mechanical properties of PGS, salt composite 3D printing technology was used to prepare PGS/PCL composite scaffolds of different proportions. The composite scaffold retains the good elasticity and fatigue resistance of PGS, while PCL can control the elastic modulus to achieve mechanical enhancement, and has the biomimetic mechanical properties of cartilage. In addition, PCL can also regulate the degradation rate and obtain the suitable regeneration and degradation cycle of trachea.

### 3D Printing of PGS/PCL Scaffolds

The composited ink was continuously extruded and printed into PGS prepolymer/salt particles hybrid constructs with multi‐layer structure by using 3D bio‐printer system (Regenovo3D Bio‐Architect). The nozzle inner diameter was 500 µm. Printing parameters of crisscrossed scaffold as follows: the center‐to‐center distance between filaments was 1.5 mm with 0°/90° lay‐down patterns between two successive layers and the height of every layer was 400 µm. The printed PGS prepolymer/salt particles constructs were then transferred to a vacuum oven and cured at 100 °C (0.5 bar) for 12 h and then further cured at 150 °C (1 bar) for 24 h. After curing, the resultant PGS scaffolds were soaked in distilled water to remove the salt for 12 h, with the water changed three times. Finally, the highly porous PGS/PCL scaffolds were freeze‐dried in vacuum.

### Fabrication of PPG Scaffolds

Gelatin of 100 mg was completely dissolved in 10 mL water. PGS scaffolds were immersed in gelatin solution for 30 min. Then they were placed in order at 4°, −20°, −80° for gradient coagulation. After coagulation for 24 h, these scaffolds were freeze‐dried in vacuum to remove water. Next, the PGS/PCL‐Gel constructs were crosslinked using EDC/NHS method for 24 h (EDC and NHS were dissolved in 98% ethanol, the concentrations of EDC and NHS were 0.5% and 0.3%, respectively). The residual solution and removed completely in deionized water. Finally, the PPG scaffolds were freeze‐dried in vacuum again.

### Optical images of PPG Scaffolds

The PPG scaffolds were dried and placed under the Light Microscope (Nikon ECLIPSE E600) at different magnifications to observe the frame structure of the PPG scaffolds and the internal gelatin honeycomb network.

### SEM of Scaffolds

PGS/PCL and PPG scaffolds were sputtered with gold and then observed in top view and sectional view at different magnifications using Field Emission Scanning Electron Microscopy (Hitachi, SU8010) with an accelerating voltage of 10 kV.

### Mechanical Properties of Scaffolds

The mechanical properties of the PGS/PCL and PPG scaffolds were analyzed using a mechanical testing machine (Instron‐5542) as reported methods. In the axial compression test, the scaffolds were compressed to maximal strain of 40% at a rate of 10 mm min^−1^ (n = 6). The moduli were calculated according to the slope of the strain–stress curves for statistical analysis. In the cyclic compression test, the specimens were compressed to a maximal strain of 40% and recovered to strain of 0% for 10 cycles at a rate of 10 mm min^−1^.

### FTIR Spectra

The chemical structure of the PGS/PCL and PPG scaffolds were analyzed by Fourier transform infrared spectroscopy (FTIR) using a Nicolet‐670 FTIR spectrometer. All spectra were recorded over a range from 1000 to 4000 cm^−1^.

### Water Contact Angle

An optical water contact angle measurement system (Dataphysics OCA20) was used to analyze the wettability characteristics of the scaffolds. Each scaffold was placed on the surface to calculate the contact angle. A droplet of deionized water was deposited on the scaffolds using a 10 µL pipette tip and a high‐resolution camera. Then, the image of the static liquid deposition was obtained within a few seconds and analyzed.

### Rabbit Chondrocytes Isolation and Culture

Both nude mice and New Zealand white rabbits were purchased from Shanghai Jiagan Experimental Animal Raising Farm. All animals were housed in isolated ventilated cages barrier facility at Shanghai Jiao Tong University Laboratory Animal Center. The mice were maintained on a 12/12‐hour light/dark cycle, 20–26 °C with sterile pellet food and water ad libitum. This study was performed in accordance with the recommendations in the Guide for the Care and Use of Laboratory Animals and relevant Chinese laws and regulations. The protocol was approved by the Institutional Animal Care and Use Committee (IACUC) of Shanghai Jiao Tong University, the Animal Protocol number was A2023069.Chondrocytes were isolated from auricular cartilage of New Zealand white rabbits. As previously reported,^[^ ^46^ ^]^ the harvested cartilage was removed fibrous tissue and perichondrium, then minced into 1 mm^2^ pieces, washed in PBS containing 1% antibiotics, and digested with 0.15% collagenase NB4 (Worthington Biochemical Corp, Freehold, New Jersey, USA) for 8 h at 37 °C. Isolated chondrocytes were cultured in Dulbecco's modified Eagle's medium (DMEM) medium (Gibco BRL, Grand Island, NY, United States) containing 10% fetal bovine serum (Gibco BRL) and 1% antibiotic‐antimycotic (Gibco BRL).

### Tissue Engineered Cartilage Regeneration In Vitro and In Vivo

A 100 µL chondrocytes suspension (80 × 10^6^ cells mL^−1^) was dropped onto the PGS/PCL‐Gel scaffolds. Subsequently, chondrocytes‐scaffolds were incubated at 37 °C for 4 h and then the culture medium was added. All cell seeded scaffolds were cultured in chondrogenic medium. After induction for 4 weeks in vitro, the engineered cartilages were harvested. After induction for 4 weeks in vitro, engineered cartilage were harvested and implanted subcutaneously into nude mice (6 weeks old, female). Nude mice were sacrificed after surgery for 8 weeks and 12 weeks implantation, and the implanted samples were collected.

### Preparation of Thermosensitive Hydrogels

To enable our patterning distribution construction, it selected an aqueous solution of Pluronic F127, a triblock copolymer with a hydrophobic poly (propylene oxide) (PPO) segment and two hydrophilic poly (ethylene oxide) (PEO) segments arranged in a PEO‐PPO‐PEO configuration, which was a relatively nontoxic non‐ionic surfactant. Aqueous Pluronic F127 triblock copolymer solutions undergo a phase transition that was both concentration and temperature dependent.^[^ ^47^, ^48^, ^49^ ^]^ In other words, a certain mass fraction of the solution can exhibit a temperature‐sensitive property–being liquid at 4 °C and gelatinous semi‐solid at 37 °C. After disinfecting Pluronic F127 with irradiation sterilization, F‐127 hydrogel of 30% mass fraction with PBS as solvent was prepared. After mixing, it was dissolved in a refrigerator at 4 °C overnight. Experiments have verified that 30% F127 hydrogel can show a temperature‐sensitive property, and it was liquid at 4 °C and semi‐solid at 37 °C, so as to realize the role of pre‐occupying during inoculating cells, so that the cell suspension can present patterning distribution.

### POS Strategy help Achieve Multi‐Cellular Precise Distribution

Temperature‐sensitive hydrogel F‐127 was first used to form a gel at a high temperature(37 °C) and occupy certain space on the PPG scaffold, then seeded the first cell suspension (chondrocytes), and then put the temperature‐sensitive hydrogel into a low temperature environment (4°) to liquefy and lose it, providing space for the seeding of the second cells. The medium was then slowly added after incubation at 37 °C for 4 h (6 well plates, 8 mL per well). After 3 days of normal culture medium, the cartilage induction medium was replaced. The culture medium was changed every two days, and the engineered cartilage was harvested after 4 weeks of induction in vitro. After the engineered cartilage matured and stabilized 4 weeks later and secreted a certain cell matrix, a second cell type (fibroblast) was seeded to achieve the patterned construction of the two cells.

### Implantation in Nude Mice

The regenerated trachea, constructed through in vitro patterning distribution, was meticulously sutured end to end using sterile absorbable sutures. Subsequently, it was implanted into subcutaneous nude mice (6‐week‐old females) with the insertion of silicone rods for additional support. The principle of surgical asepsis was strictly adhered to throughout the entire procedure: First, the dorsal area of nude mice was thoroughly disinfected using 75% alcohol. Subsequently, tissue scissors were employed to delicately create a small incision (≈2 cm) on the back of nude mice, followed by implantation of the harvested regenerated trachea into their dorsum. Intermittent suturing with absorbable sutures and subsequent disinfection were performed accordingly. The nude mice were housed in cages according to groups and provided with bedding, water, and feed for maintenance. At 8 and 12 weeks post‐surgical implantation, euthanasia was conducted on the nude mice, and samples from the implanted site were collected promptly and soaked in saline solution to maintain their viability. Finally, photographic documentation of the engineered cartilage's appearance was captured using a camera.

### Extraction and Expansion of Airway Epithelial Basal Cells

The tracheal epithelial basal cells were isolated from New Zealand rabbits by harvesting 1–2 segments of the trachea, removing fibrous tissue, isolating endo‐airway epithelium, chopping them into 1 mm^2^ small pieces, thoroughly washing them in PBS containing 1% antibiotics, and digesting them with 0.15% collagenase NB4 (Worthington Biochemical Corp, Freehold, New Jersey, USA) at 37 °C for 2 h. Subsequently, the digested fluid was filtered using a filter to obtain the airway basal cells through suspension centrifugation. These isolated cells were then cultured in specialized EX‐plus medium (STEMCELL) designed for epithelium growth. Once reaching 90% confluence, the second‐generation airway basal cells were selected for subsequent experiments.

### Histological and Immunohistochemical Analyses

The regenerated trachea specimens were fixed in 4% paraformaldehyde, embedded in paraffin, and sectioned into 5‐µm sections. All samples were stained with HE, SO/FG, and Masson as described previously. Expression of collagen II, a rabbit anti‐human monoclonal antibody against COL‐II was used with a horseradish peroxidase (HRP)‐conjugated anti‐rabbit antibody (1:400 in PBS, Santa Cruz) as the secondary antibody. The SO/FG‐stained images were further analyzed to evaluate regenerated tissue percentage by ImageJ software. The tissue sections were immunohistochemically treated with anti‐vWF and *α*‐SMA kits, and the nuclei were stained with DAPI. The expression of vWF and *α*‐SMA was observed under the confocal microscope, and the optical density of the photos was measured by ImageJ to evaluate the angiogenesis of the tissues after stent implantation.

### Quantitative Analysis

Quantitative analysis of regenerated cartilage in vitro and in vivo was performed as described previously. GAG, DNA and Total collagen content in the samples (n = 4) were respectively quantified via an Alcian Blue method, a Pico Green dsDNA assay (Invitrogen), and a hydroxyproline assay kit (Sigma–Aldrich). The regenerated cartilages were compressed to maximal strain of 20% at a rate of 10 mm min^−1^ (n = 6). The moduli were calculated by taking the stress–strain curve. Data were analyzed with Origin software. All values were reported as a mean ± standard deviation. *p < 0.05 were considered significant.

### Confocal Microscopy Verifies the Accuracy of the Distribution

Chondrocytes and fibroblasts were stained with two fluorescent probes, Dio (a green fluorescent probe for cell membrane) (C1038) and Dil (a red fluorescent probe for cell membrane) (C1036), respectively. After the two types of cells were seeded on the PGS/CS bioactive scaffold using a temperature‐sensitive hydrogel sacrificially, the cell distribution was observed under fluorescence confocal microscopy (Niko).

### Mechanical Properties of MI‐TET

The mechanical properties of the MI‐TETs, pure PGS tubular scaffolds, and natural trachea were analyzed using a mechanical testing machine (Instron‐5542). Prior to the test, the four sample tubes were placed in PBS at room temperature for equilibration. In the axial compression test, the tubular samples were compressed to a maximum strain of 40% at a rate of 10 mm min^−1^ (n = 6), and the compressive loads under 20% and 40% strains were selected for statistical analysis. In the cyclic compression test, the sample was also compressed to a maximum strain of 40% at a rate of 10 mm min^−1^, then recovered to a strain of 0%, with each cycle repeated ten times. The original values were recorded and used to plot the compressive stress curve.

### Airway Epithelium Staining and Analysis

The reconstructed sections of airway epithelium were stained with immunofluorescence by ELISA kit, and the airway epithelium markers PCK and CK‐5 were stained and labeled. The nucleus was stained by DAPI. Confocal fluorescence microscopy was used to photograph the PCK and CK5‐positive epithelial cell structures in the images, and Image J software was used for image analysis to evaluate the reconstruction of airway epithelium.

### Statistical Analysis

Data (n = 4) were expressed as the means ± standard deviations. A one‐way analysis of the variance was used to determine the statistical significance of the difference between groups using GraphPad Prism 7.0 software, and a *P*‐value < 0.05 was considered statistically significant.

## Conflict of Interest

The authors declare no conflict of interest.

## Supporting information

Supporting Information

Supplemental Movie 1

Supplemental Movie 2

Supplemental Movie 3

Supplemental Movie 4

Supplemental Movie 5

Supplemental Movie 6

## Data Availability

Data sharing is not applicable to this article as no new data were created or analyzed in this study.
