# Supplementary material for: Multi‐Tissue Integrated Tissue‐Engineered Trachea Regeneration Based on 3D Printed Bioelastomer Scaffolds
Source: Adv Sci (Weinh). 2024 Aug 19;11(39):2405420. doi: 10.1002/advs.202405420 (PMC11497002; doi:10.1002/advs.202405420)
Supplement: Supplementary file 1 — Supporting Information [file ADVS-11-2405420-s003.docx]

Supplementary Materials for

**Multi-tissue Integrated Tissue-engineered Trachea Regeneration Based on 3D Printed Bioelastomer Scaffolds**

Xingqi Song, Peiling Zhang, Bin Luo, Ke Li, Yu Liu, Sinan Wang, Qianyi Wang, Jinyi Huang, Xiaohong Qin, Yixin Zhang^*^, Guangdong Zhou^*^ and Dong Lei^*^.

Xingqi Song, Peiling Zhang, Ke Li, Yu Liu, Sinan Wang, Qianyi Wang, Jinyi Huang, Yixin Zhang, Guangdong Zhou, Dong Lei.

Department of Plastic and Reconstructive Surgery, Department of Cardiology, Shanghai Key Lab of Tissue Engineering, Shanghai 9th People’s Hospital, Shanghai Jiao Tong University School of Medicine, Shanghai, 200011, P.R. China

E-mail: zhangyixin6688@163.com (Y.Z.); guangdongzhou@126.com (G.Z.); leidongjesse@qq.com (D. L.)

Bin Luo, Xiaohong Qin.

College of Textiles, State Key Laboratory for Modification of Chemical Fibers and Polymer Materials, Donghua University, Shanghai 201620, P. R. China.

**This PDF file includes:**

Supplementary Text

Figure S1 to S5#

Table 1 and 2

**Other Supplementary Materials for this manuscript include the following:**

Movie S1 to S6#

Supplementary Text

**Supplementary scaffold hydrophilicity tests**

The modification of gelatin resulted in increased hydrophilicity of the PPG scaffolds. Upon water absorption, the weight increase of PPG scaffolds (1040.67 ± 111.73 mg) was significantly higher than that of PGS/PCL scaffolds (154.33 ± 3.21 mg) (**Figure S1**A). In the dynamic water contact angle experiment, the contact angle between PPG scaffolds and water droplet reached 0 within 10 seconds (complete absorption), while the contact angle in the PGS/PCL scaffold group remained at 61 degrees even after 5 minutes (incomplete absorption). These findings suggest that gelatin-modified PPG scaffolds exhibit more superior hydrophilicity and biological activity, making them more suitable for cell adhesion and proliferation.

**Precise patterned distribution and sacrifice of thermo-sensitive hydrogels**

We first developed a Post-Occupancy Sacrifice (POS) strategy of thermosensitive hydrogel to facilitate a patterned construction of multiple cells. We selected Pluronic F-127 with 30% mass fraction, which exhibits a temperature-sensitive property -- being liquid at 4 °C and gelatinous semi-solid at 37 °C. This gelatinous semi-solid can effectively impede the distribution of cells on our scaffolds, and subsequently transition into a liquid state and dissipate at 4 °C, so that it plays a role of temporary obstructive barrier during cell inoculating. Through this POS strategy, the precise distribution of complex patterns (such as “SJTU”) can be easily achieved (**Figure S2**).

**Histological staining of the regenerative integrated trachea for 8/12 weeks**

To evaluate the maturity of the implanted tracheal cartilage and determine if the in vitro multi-tissue patterning structure can be maintained after in vivo culture, histological staining was performed on two sections of the regenerative integrated trachea, the radial and axial directions. The radial section corresponds to the C-shaped cartilage ring (C ring) and O-shaped fiber ring (F ring) of the trachea, while the axial section corresponds to the longitudinal cartilage/fiber interleaving area (C/F band) and the strip fiber bundle area (F bundle), respectively. In the main text, we showed the cartilage-specific SO/FG staining results and the immunofluorescence results of vascularization indicators vWF and α-SMA. In order to show the tissue maturity of the regenerative integrated trachea more comprehensively, additional staining techniques including HE (**Figure S3**), Masson (**Figure S4**), and Col II (type II collagen) (**Figure S5**) staining were displayed. These results further confirmed the formation of chondroid tissue in the radial C-shaped cartilage area, which exhibited typical lacunar-like structures and extensive ECM deposition. The axial cross section of the cartilage/fiber still maintained the alternating arrangement of cartilage tissue and fiber tissue. The section of the axial fiber bundle was mostly covered by fiber tissue. With the verification of multiple histological stains, it turned out that our regenerative integrated trachea successfully maintains the original structural design in nude mice.


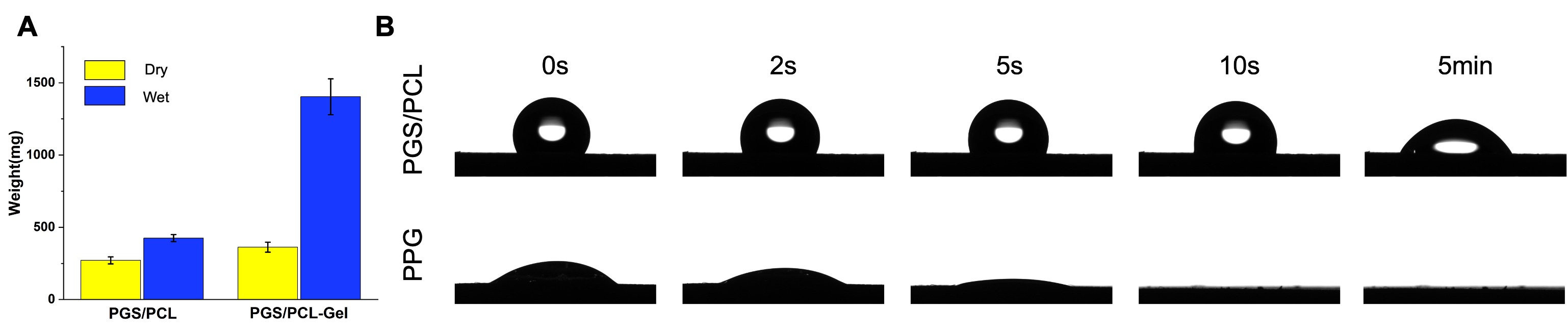


Figure S1.

Hydrophilicity of the scaffolds. A) Mass growth of two scaffolds after water absorption. B) Water contact angle tests of PGS/PCL and PPG scaffolds from 0s to 5min.


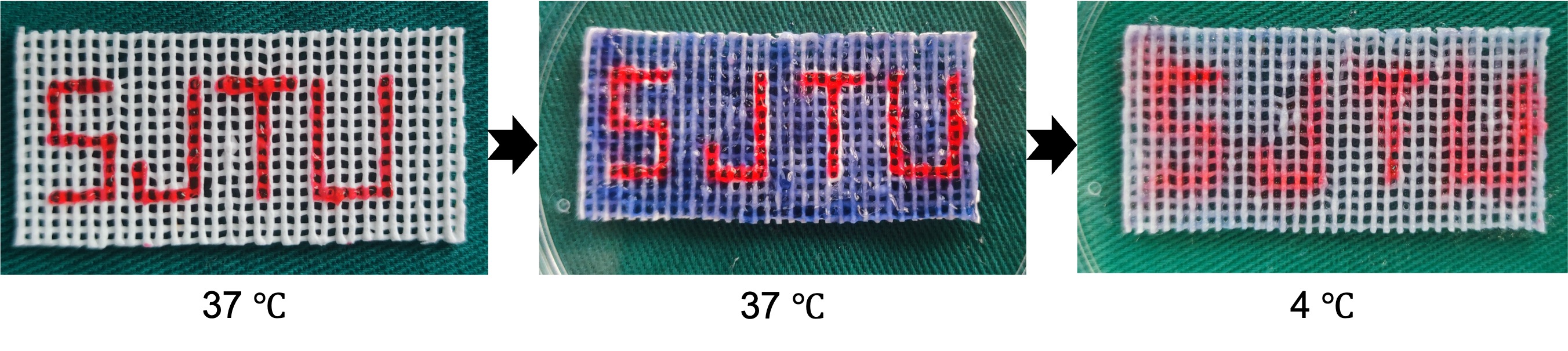


Figure S2.

The precise distribution of the thermo-sensitive hydrogel on the scaffold at a low temperature (4 ℃), and its liquefication and sacrifice characteristics are demonstrated at a high temperature (37 ℃)


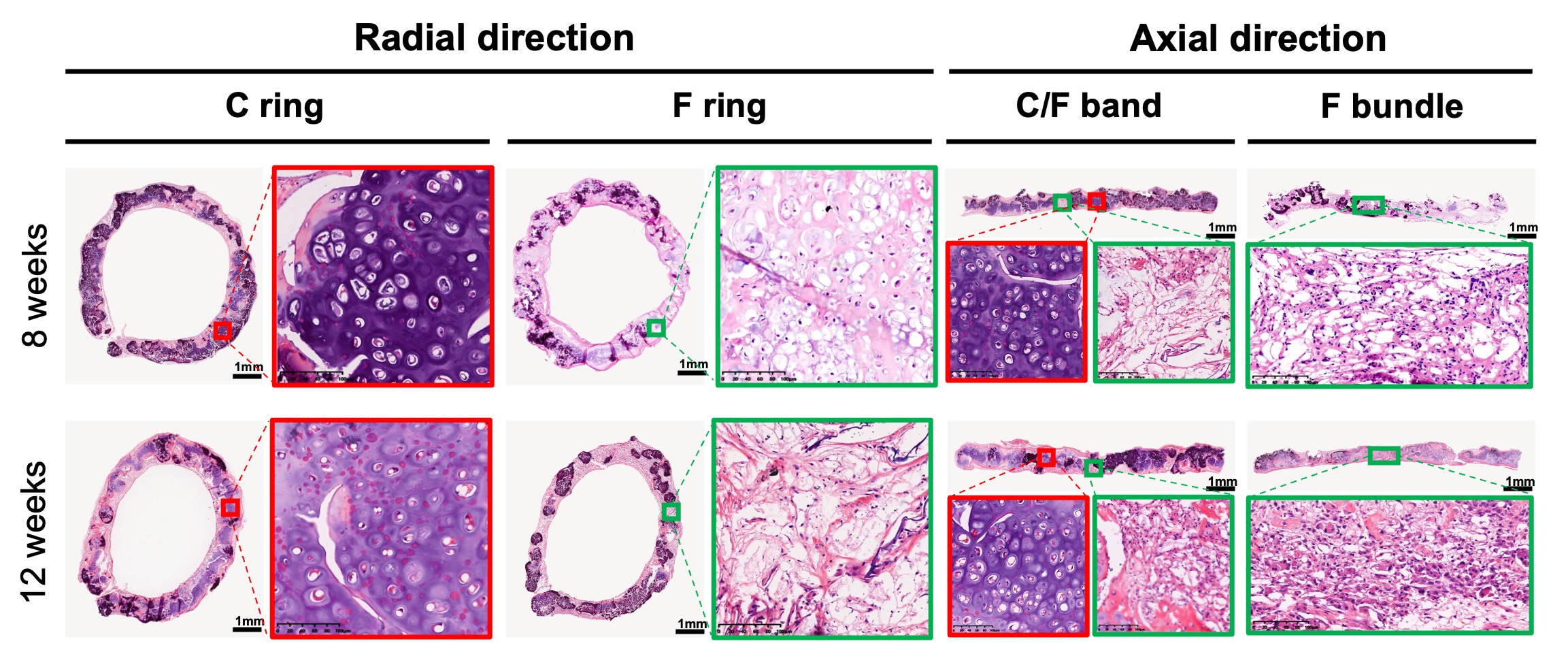


Figure S3.

Results of HE staining of the regenerative integrated trachea for 8/12 weeks from four sections (C ring and F ring section in radial direction; C/F band and F bundle section in axial direction). Red rectangles represent cartilage regions; green rectangles represent vascularized fibrous tissue regions; Scale bar: 1 mm, 100 μm.


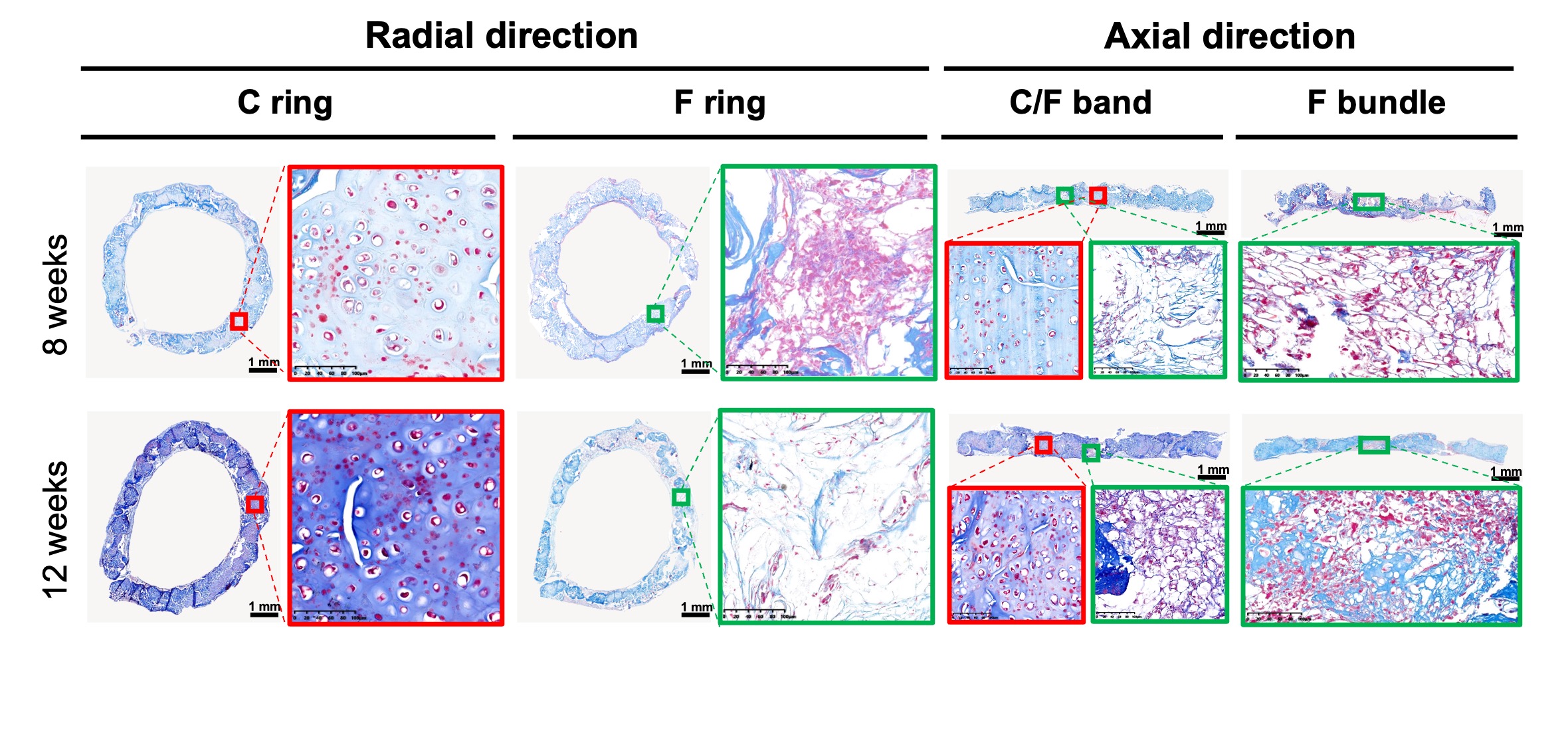


Figure S4.

Results of Masson staining of the regenerative integrated trachea for 8/12 weeks from four sections (C ring and F ring section in radial direction; C/F band and F bundle section in axial direction). Red rectangles represent cartilage regions; green rectangles represent vascularized fibrous tissue regions; Scale bar: 1 mm, 100 μm.


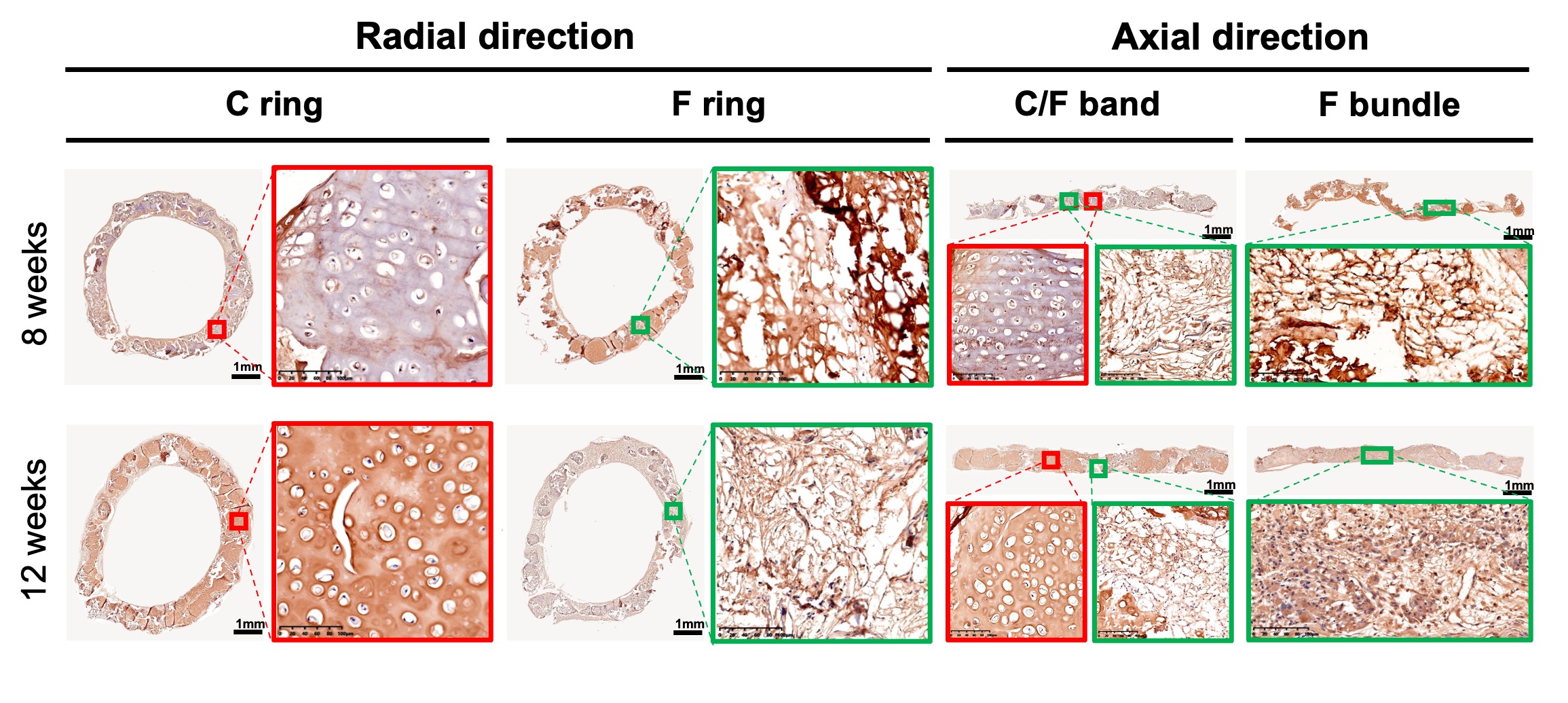


Figure S5.

Results of COL II staining of the regenerative integrated trachea for 8/12 weeks from four sections (C ring and F ring section in radial direction; C/F band and F bundle section in axial direction). Red rectangles represent cartilage regions; green rectangles represent vascularized fibrous tissue regions; Scale bar: 1 mm, 100 μm.

|  | X-axis：Evaluation of functional reconstruction | | | | | |
| --- | --- | --- | --- | --- | --- | --- |
|  | GAG | Collagen | Modulus | Vascularization | Epithelialization | Total |
| Natural  trachea | 20 | 20 | 20 | 20 | 20 | 100 |
| This work | 20 | 20 | 20 | 15 | 15 | 90 |
| 【39】 | 20 | 20 | 20 | 0 | 0 | 60 |
| 【40】 | 20 | 20 | 20 | 0 | 0 | 60 |
| 【30】 | 15 | 14 | 16 | 0 | 0 | 45 |
| 【41】 | 20 | 20 | 20 | 0 | 10 | 70 |
| 【10】 | 18 | 20 | 14 | 15 | 10 | 77 |
| 【11】 | 20 | 20 | 18 | 5 | 10 | 73 |
| 【7】 | 16 | 18 | 16 | 20 | 18 | 88 |
| 【9】 | 20 | 20 | 7 | 10 | 0 | 57 |
| 【14】 | 18 | 18 | 14 | 0 | 15 | 65 |
| 【15】 | 20 | 19 | 20 | 10 | 0 | 69 |
| 【21】 | 20 | 20 | 10 | 20 | 15 | 85 |

Table S1. Evaluation of functional reconstruction of TET

In evaluating tracheal function, we focused on five key indicators: GAG, collagen, modulus, vascularization level, and epithelization degree. We give each indicator 20 points for a total of 100 points. Our analysis of the research literature revealed varying scores across these indicators. While some studies excelled in basic properties such as GAG, collagen, and elastic modulus, others showed significant deficiencies in vascularization level and epithelialization degree, resulting in lower scores. This study on multi-tissue integrated trachea outperformed all previous tracheal regeneration studies in terms of tracheal function, approaching the functionality of a natural trachea.

|  | Y-axis：Fourfold tissue structure pre-build | | | | |
| --- | --- | --- | --- | --- | --- |
|  | C-shaped Cartilage ring | O-shaped vascularized ring | Fiber bundle | Airway epithelium | Total |
| Natural  trachea | 25 | 25 | 25 | 25 | 100 |
| This work | 25 | 25 | 25 | 25 | 100 |
| 【39】 | 0 | 0 | 0 | 0 | 0 |
| 【40】 | 0 | 0 | 0 | 0 | 0 |
| 【30】 | 0 | 0 | 0 | 0 | 0 |
| 【41】 | 25 | 0 | 0 | 25 | 50 |
| 【10】 | 0 | 25 | 0 | 0 | 25 |
| 【11】 | 0 | 0 | 0 | 0 | 0 |
| 【7】 | 0 | 25 | 0 | 0 | 25 |
| 【9】 | 0 | 25 | 0 | 0 | 25 |
| 【14】 | 0 | 0 | 0 | 0 | 0 |
| 【15】 | 25 | 25 | 25 | 0 | 75 |
| 【21】 | 25 | 25 | 25 | 0 | 75 |

Table S2. Evaluation of Fourfold tissue structure pre-build

In the realm of four-fold tissue structure construction, we give 25 points for each prebuild of the four organizational structures, for a total of 100 points. Previous studies have given minimal attention to the intricate multi-tissue composition of the trachea. Only a handful of papers have successfully engineered trachea tissue regeneration in vitro that aligns with the natural physiological structure of the trachea. This study, for the first time, achieved the integrated construction of a four-fold tissue structure in vitro, effectively mimicking the C-shaped cartilage ring, O-shaped vascularized fiber ring, strip fiber bundle, and airway epithelium. This breakthrough introduces a novel concept for the future advancement of tissue-engineered trachea.

Movie S1.

Process of 3D printing PGS scaffolds.

Movie S2.

Dynamic contact angle test of PGS/PCL.

Movie S3.

Dynamic contact angle of PPG scaffolds.

Movie S4.

Regenerative integrated trachea implanted subcutaneously in nude mice.

Movie S5.

Mechanical display of 8-week regenerated trachea.

Movie S6.

Mechanical display of 12-week regenerated trachea.
